# A modified clot-based assay to measure negatively charged procoagulant phospholipids

**DOI:** 10.1038/s41598-021-88835-y

**Published:** 2021-04-29

**Authors:** Cathrine Ramberg, S. Jamaly, N. Latysheva, L. Wilsgård, T. Sovershaev, O. Snir, J.-B. Hansen

**Affiliations:** 1 K.G. Jebsen Thrombosis Research and Expertise Centre (TREC), Department of Clinical Medicine, UiT—The Arctic University of Norway, 9037 Tromsø, Norway; 2 Division of Internal Medicine, University Hospital of North Norway, Tromsø, Norway

**Keywords:** Diseases, Medical research

## Abstract

Growing evidence supports a role for extracellular vesicles (EVs) in haemostasis and thrombosis due to exposure of negatively charged procoagulant phospholipids (PPL). Current commercial PPL-dependent clotting assays use chemically phospholipid depleted plasma to measure PPL activity. The purpose of our study was to modify the PPL assay by substituting the chemically phospholipid depleted plasma with PPL depleted plasma obtained by ultracentrifugation This in order to get readily access to a sensitive and reliable assay to measure PPL activity in human plasma and cell supernatants. The performance of the assay was tested, including the influence of individual coagulation factors and postprandial lipoproteins and compared to a commercial PPL assay (STA-Procoag-PPL). The two PPL assays displayed similar sensitivity to exogenously added standardized phospholipids. The PPL activity measured by the modified assay strongly correlates with the results from the commercial assay. The intraday- and between-days coefficients of variation ranged from 2–4% depending on the PPL activity in the sample. The modified PPL assay was insensitive to postprandial lipoprotein levels in plasma, as well as to tissue factor (TF) positive EVs from stimulated whole blood. Our findings showed that the modified assay performed equal to the comparator, and was insensitive to postprandial lipoproteins and TF^+^ EVs.

## Introduction

Procoagulant phospholipid (PPL) activity has regained interest in recent years, mainly due to the increased understanding of the role of extracellular vesicles (EVs) in thrombosis and haemostasis^1,2^. As early as 1946, Chargaff and West observed that the clotting time of plasma was prolonged by applying high-speed centrifugation to remove “thromboplastic substances”^3^. Accordingly, Connor and colleagues demonstrated that the amount of annexin A5 positive EVs, measured by flow cytometry, showed a significant and inverse correlation with clotting time^4^. These findings suggest that EVs play a significant role in coagulation, apparently due to exposure of phospholipids, and phosphatidylserine (PS) in particular, on their surface. The increase in surface expression of negatively charged PPL will facilitate the assembly of coagulation factors upon cell activation or apoptosis^5^. This is crucial for several stages of the coagulation pathway, namely the formation of the intrinsic and extrinsic tenase complexes, as well as the conversion of prothrombin to thrombin by coagulation factor Xa (FXa)^6^. The activity of the extrinsic tenase complex, the tissue factor (TF)—factor VIIa (FVIIa) complex, is increased by several orders of magnitude in the presence of negatively charged membrane phospholipids^7^.

Several assays have been developed to measure the PPL activity in human plasma. While some are based on the ability of annexin A5 to bind PS in the presence of Ca^2+^^4,8^, others are clot-based, utilizing the ability of PPL to accelerate the conversion of prothrombin to thrombin. Annexin A5-based assays are widely used, often in a flow cytometry setting, a method that is time consuming, requires expensive equipment and experienced personnel. In addition, annexin A5 is commonly used in chromogenic FXa assays, where the activity measured is based on the EVs exposing PS that are bound to the microplate. These EVs are then able to accelerate the cleavage of the chromogenic substrate by FXa^9^.

Compared to FXa chromogenic assays, which measure procoagulant activity of EVs in a purified system, clotting assays involve a more complex reaction and a physiological end-point as they measure the PPL activity of plasma, and not only captured PS-positive EVs^9^. To the best of our knowledge, there are currently two clot-based assays commercially available, the STA-Procoag-PPL assay from Diagnostica Stago (Asnières sur Seine Cedex, France) and the XACT assay from Haematex (Hornsby, NSW, Australia). Both assays use chemical phospholipase treatment to deplete phospholipids from plasma, but differ with regard to the phospholipase used, plasma origin, and the use of a phospholipid calibrator. The XACT assay uses a snake phospholipase^10^ and porcine plasma, while an unspecified phospholipase and human plasma is used for the Stago assay.

In this study, we aimed to develop a modified PPL-dependent clotting assay, capable of measuring the PPL activity in human plasma and cell supernatants of in vitro experiments, by removing PPL from plasma by sequential centrifugation, including final ultracentrifugation. The performance of the modified assay was then validated against the commercially available Stago STA-Procoag-PPL assay.

## Results

### Impact of stepwise EV depletion on PPL clotting times

**Figure 1:**
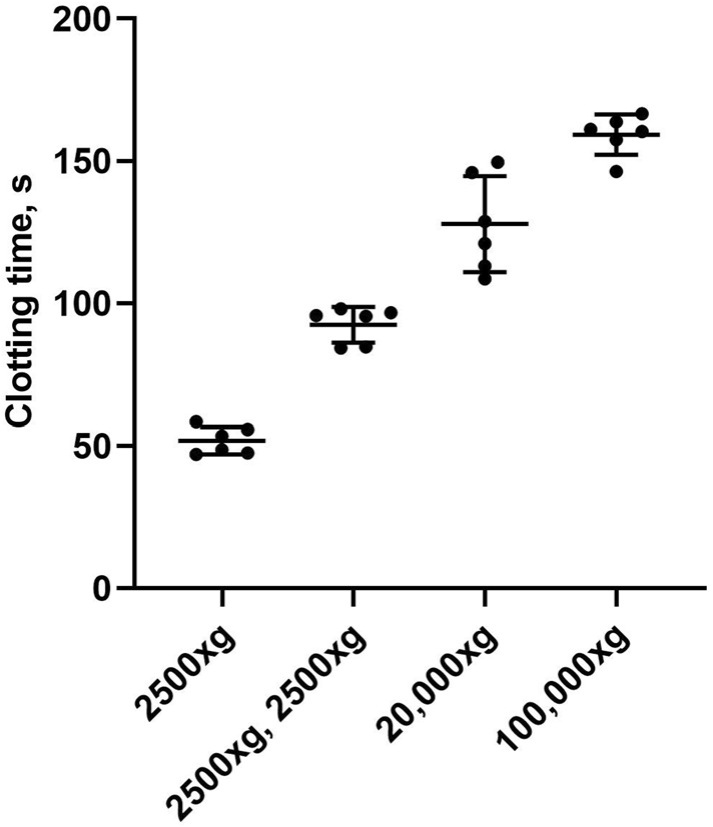
The effect of sequential centrifugation on plasma PPL clotting times. Citrated blood collected from 6 healthy volunteers was subjected to consecutive centrifugations: 2500×*g* for 15 min to obtain platelet poor plasma (PPP), 2500×*g* for 15 min to obtain platelet free plasma (PFP), 20,000×*g* for 30 min and 100,000×*g* for 1 h to deplete for EVs. A sample of plasma after each centrifugation was analyzed with the modified PPL assay. The PPL activity is presented in seconds (s) of clotting time. Dot plot with mean ± 1 SD (n = 6 for each condition).

The removal of PPL from plasma is fundamental for the clot-based assays. As EVs are the main source of PPL, we first tested whether sequential centrifugation reliably depleted EVs from plasma. To achieve this, we compared the PPL clotting times (PPL_CT_) of plasma samples (n = 6) subjected to sequential centrifugation procedures (Fig. 1). Plasma prepared by centrifugation at 2500×*g* for 15 min caused clotting times of 51.8 ± 4.7 s (mean ± 1 SD). A second centrifugation step of 2500×*g* for 15 min resulted in a prolongation of the clotting times to 92.5 ± 6.3 s (mean ± 1 SD). Pelleting larger EVs (e.g. microvesicles) from platelet free plasma (PFP) by an additional spin of 20,000×*g* for 30 min at room temperature (RT) further prolonged the clotting times to 127.8 ± 16.9 s (mean ± 1 SD). The final spin at 100,000×*g* for 60 min to remove the smallest and lightest EVs (e.g. exosomes) further prolonged the clotting times to 159.3 ± 7.1 s (mean ± 1 SD).

Procoagulant phospholipid depleted plasma (PPL depleted plasma) prepared from pooled PFP (2500×*g* for 15 min twice) by ultracentrifugation (100,000×*g* for 60 min) resulted in a mean clotting time of 163.2 ± 7.5 s (mean ± 1 SD) (Supplementary Figure S1).

### PPL depletion by ultracentrifugation does not affect standard coagulation assays

To test whether the preparation of PPL depleted plasma affected standard coagulation assays, we measured activated partial thromboplastin time (aPTT) and prothrombin time (PT). The aPTT was 30.2 (normal range 25–37 s) and PT 21.3 s, corresponding to a PT-INR of 1.00 (normal range < 1.1).

### Comparison of the modified PPL assay and the STA-Procoag-PPL assay

**Figure 2:**
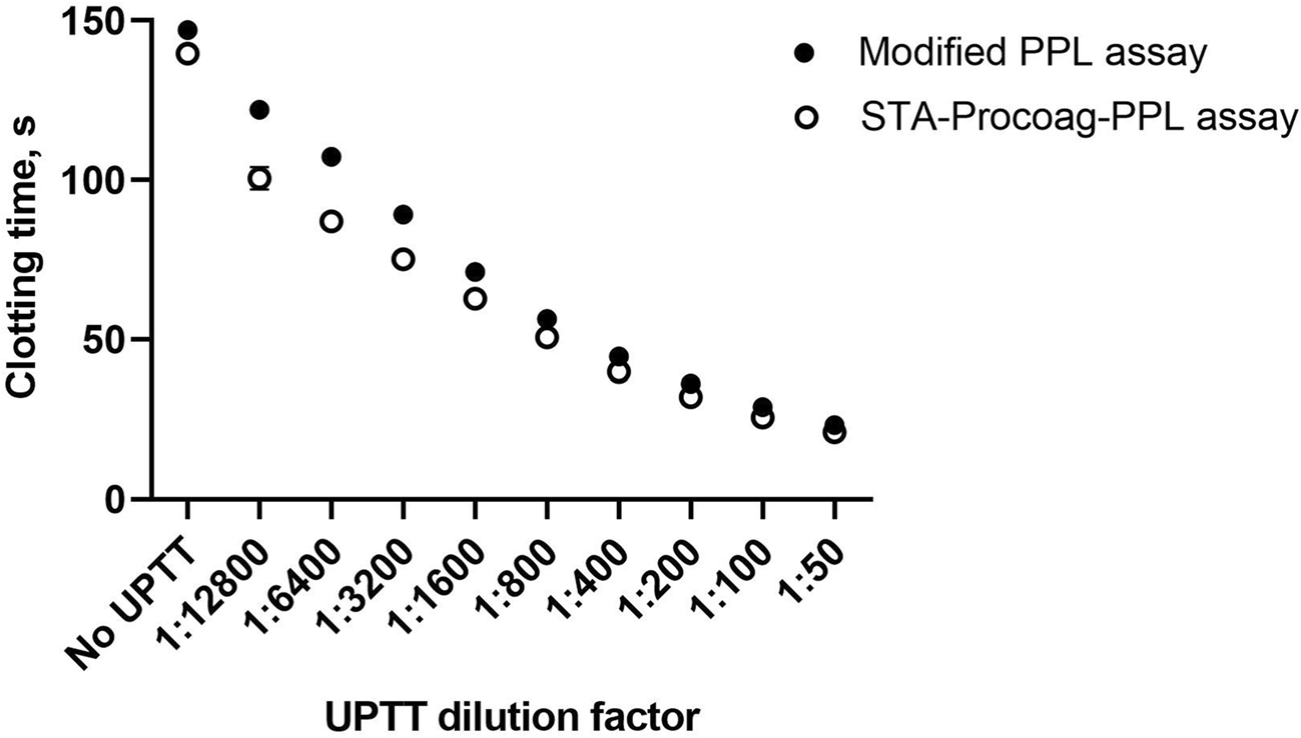
Comparison of the clotting times obtained by the STA-Procoag-PPL and the modified PPL assays using a standardized phospholipid reagent (UPTT reagent). Serial dilutions of UPTT (from 1/50 to 1/12,800), was added to PPL depleted plasma and tested on the modified PPL assay (closed circles) and the STA- Procoag-PPL assay (empty circles). Values are means of three experiments ± 1 SD.

In order to demonstrate that PPL depleted plasma prepared by ultracentrifugation was comparable to enzymatic depletion of PPL, we tested the sensitivity of the STA-Procoag-PPL assay and the modified PPL assay using serial dilutions of a standardized phospholipid reagent (UPTT reagent). The addition of UPTT shortened the clotting times in a concentration dependent manner (Fig. 2). The two assays performed similarly throughout the tested concentration range. Dilution curves of bovine FXa (bFXa) tested in PPL depleted plasma and pooled PFP supported the use of 0.1 U/ml bFXa for the modified PPL assay (Supplementary Figure S2). Despite a ten-fold lower concentration of bFXa used in the STA-Procoag-PPL assay, it consistently displayed shortened clotting times compared to the modified assay.

**Figure 3:**
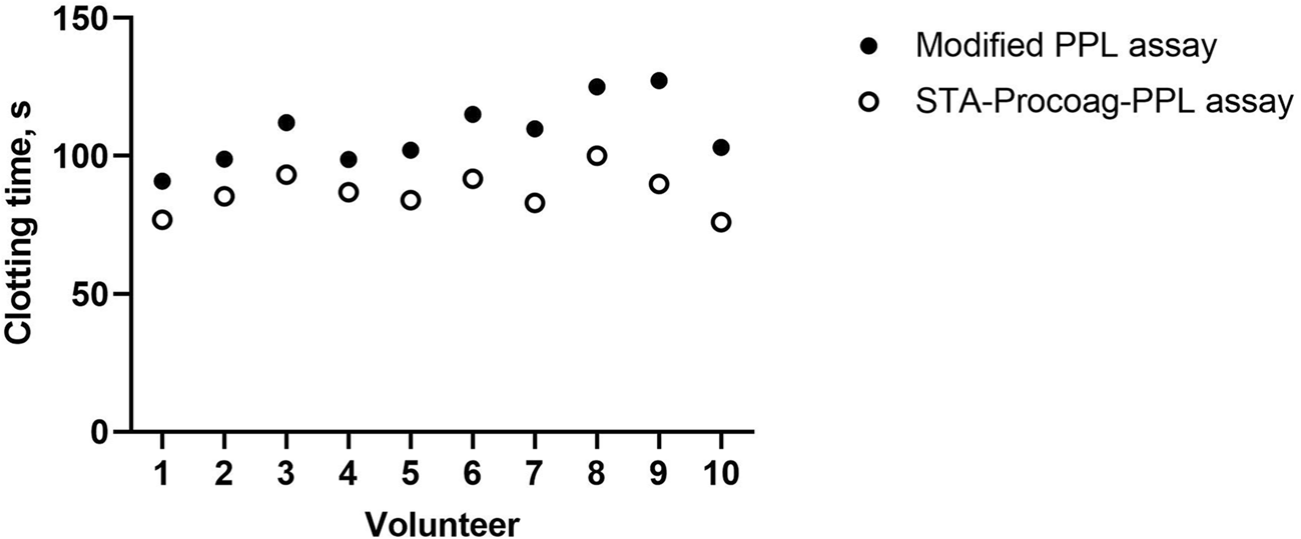
Comparison of PPL clotting times (PPL_CT_) measured by the modified PPL assay (closed circles) and the STA-Procoag-PPL kit (empty circles). Platelet free plasmas from 10 healthy individuals were tested on both assays and the PPL_CT_ is presented in seconds (s). Values are mean of duplicate measurements.

To compare the performance of the modified PPL and STA-Procoag-PPL assays, plasma samples from ten healthy blood donors were tested on both assays (Fig. 3). A strong correlation (r = 0.76, p < 0.01) was found between the assays, confirming their comparable performance. PPL_CT_ measured by the modified assay were consistently longer than for the STA-Procoag-PPL assay.

### Assay reproducibility

To assess the coefficients of variation (CV) of the modified PPL assay, we used the two plasma standards provided by the STA-Procoag-PPL assay, one resulting in PPL_CT_ of 16 s. and the other PPL_CT_ of 54 s, as well as a pooled PFP sample with PPL_CT_ of 85 s. The day-to-day CV for the Stago standards (n = 30) was 3.9% and 3.2%, respectively, whereas the within-a-day CV (n = 20) was 3.7% and 2.8%, respectively. Pooled PFP had a day-to-day CV of 4.1% and a within-a-day CV of 3.3%.

The reproducibility of three independent preparations of PPL depleted plasma from the same six donors was tested, and demonstrated highly comparable PPL_CT_ measurements without the addition of UPTT and with the addition of two different concentrations of UPTT (PPL depleted plasma without UPTT: 163.2 ± 7.5 s, 1:3200 U/ml of UPTT: 102.3 ± 5.7 s, 1:100 U/ml of UPTT: 30.6 ± 0.9 s) (Supplementary Figure S1).

### *Impact of different PFP preparation protocols on PPL*_*CT*_

**Figure 4:**
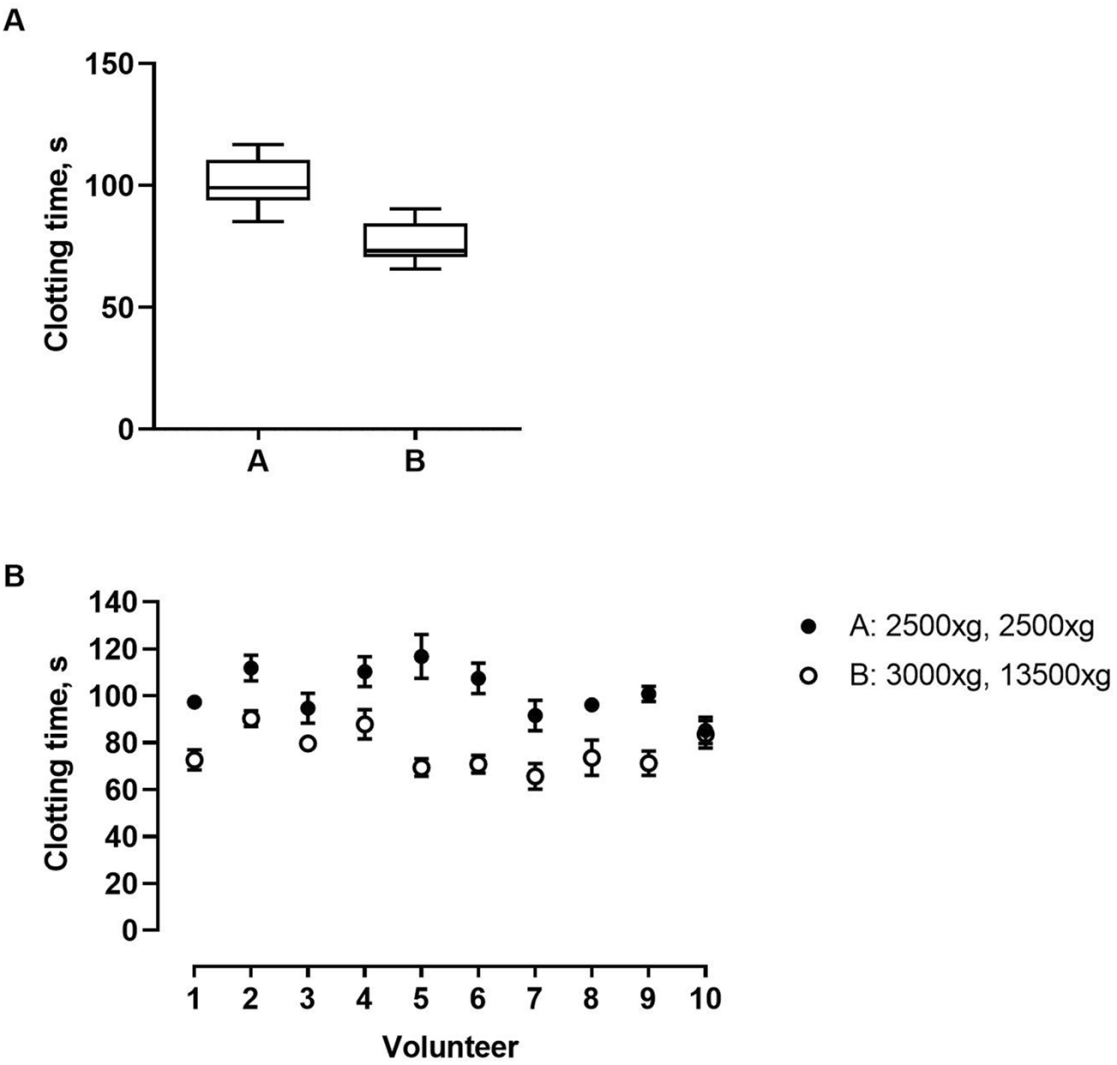
The effect of platelet free plasma (PFP) preparation on clotting time. PFP from 10 volunteers were prepared using two different centrifugation protocols, (**A**) 2500×g for 15 min twice or (B) 3000×*g* for 10 min followed by 13,500×*g*. Clotting times were measured using the modified PPL assay. Panel (**A**) is a box plot of all ten volunteers combined for the two protocols, while panel (**B**) displays the individual values as mean of duplicate measures ± 1 SD.

We wanted to investigate to what extent two different centrifugation protocols (Protocol A: 2500×*g* for 15 min twice; protocol B: 3000×*g* for 10 min followed by 13,500×*g* for 2 min) affected PPL_CT_ (Fig. 4). Plasmas obtained by protocol A from 10 healthy individuals displayed prolonged PPL_CT_ compared to plasmas obtained by protocol B (101.2 ± 10.9 s versus 76.5 ± 9.1 s, p < 0.0001).

### *Postprandial lipemia does not affect PPL*_*CT*_* in the modified PPL assay*

**Figure 5:**
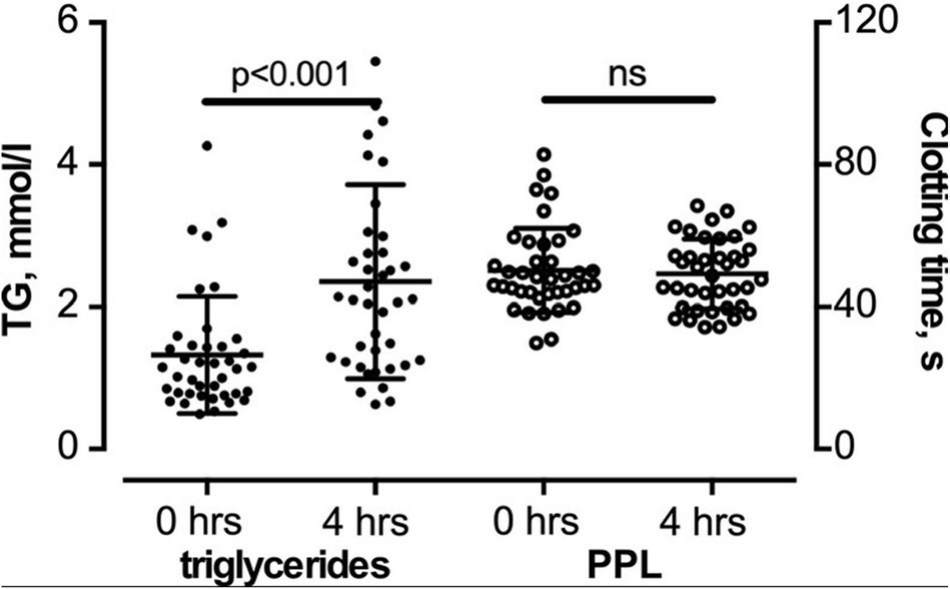
The effect of postprandial lipemia on plasma PPL activity. Serum triglycerides increased significantly after the ingestion of the standardized high fat meal without affecting the PPL activity. Dot plots are means ± 1 SD, n = 40 for each group.

To test the sensitivity of the modified PPL assay to postprandial lipemia, we compared the PPL_CT_ of PFP prepared from blood collected before (0 h) and 4 h after a standardized high fat meal (1 g fat/kg body weight) (Fig. 5). This caused a prompt increase in total serum triglycerides that peaked after 4 h and almost reached baseline concentrations 8 h after the meal^11^. However, postprandial lipemia was not accompanied by a significant change in the PPL_CT_ of fasting and postprandial plasma samples (51.1 ± 12.4 s and 48.9 ± 9.3 s, respectively). This indicates that the modified PPL assay was insensitive to postprandial lipemia measured in PPP samples subjected to a second high speed centrifugations after thawing, that generate mean CT values around 50 s.

### The impact of FVIIa, FVa and surface PS on the modified PPL assay

**Figure 6:**
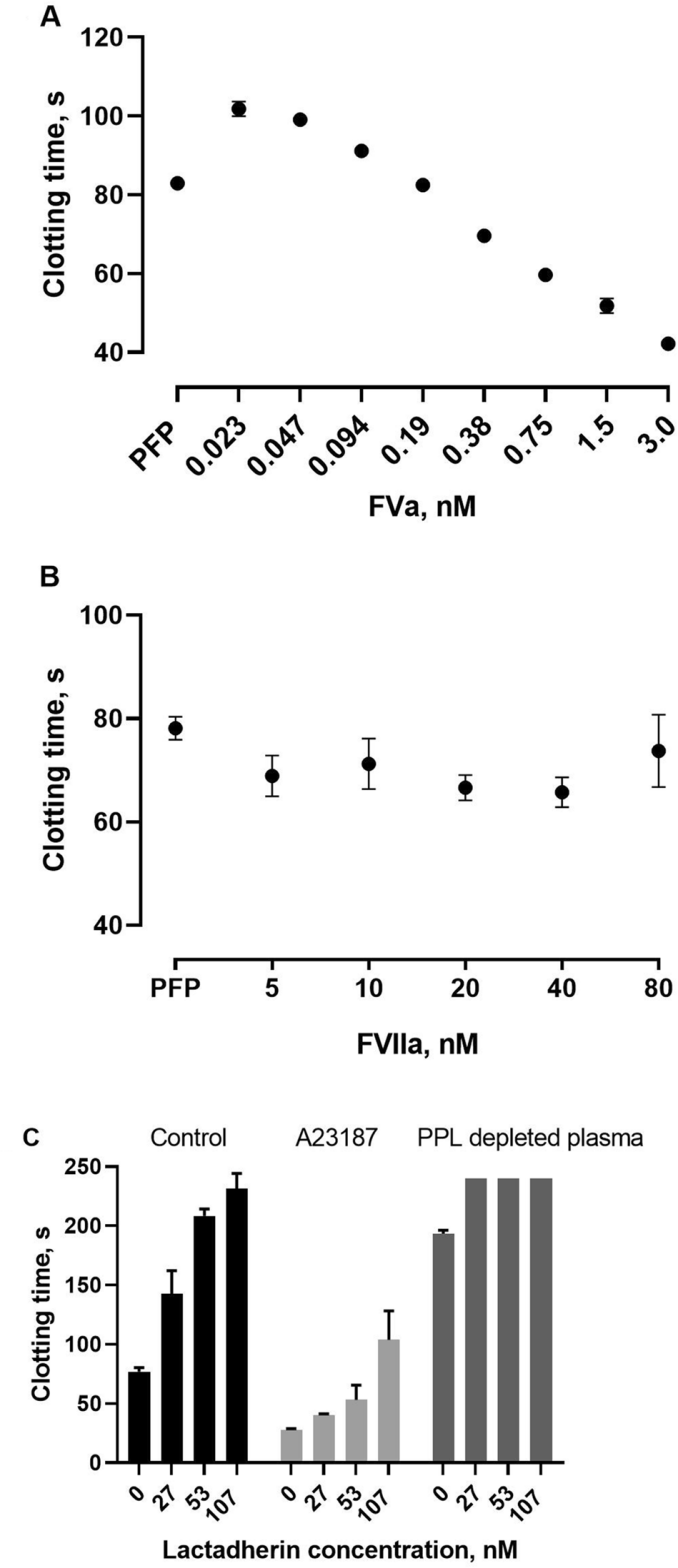
The effect of coagulation factors on the performance of the modified PPL assay. Exogenous coagulation factors were added to the reaction buffer in concentrations from 0.02 to 3.0 nM for FVa (panel **A**) and from 5 to 80 nM for FVIIa (panel **B**). Panel (**C**) shows the effect of lactadherin pretreatment on the PPL activity of PRP-derived EVs generated by stimulation of PRP by calcium ionophore A23187 for 15 min. Values are means ± 1 SD (n = 4).

Since the presence of coagulation factors in human PPL depleted plasma may provide a source of preanalytical variation of the modified PPL assay, we tested the assay with the addition of various concentrations of coagulation factors V/Va and VIIa (Fig. 6). The addition of FVa resulted in a dose dependent shortening of PPL_CT_ (Fig. 6A). The addition of increasing levels of FVIIa had minor influence on PPL_CT_, although the initial addition of 5 nM FVIIa slightly shortened the PPL_CT_ (Fig. 6B).

To further prove that coagulation in the modified assay is mainly driven by PPL, we used lactadherin to neutralize the negatively charged phospholipids. Pretreatment with lactadherin reduced the PPL_CT_ dose-dependently of EVs derived both from A23187-stimulated PRP and EVs from unstimulated PRP (Fig. 6C). Pretreatment of both PPL depleted plasma and PFP with 107 nM lactadherin drastically reduced their PPL_CT_ with clotting times exceeding the maximum clotting time of the coagulometer.

### *Blood-borne TF has no effect on PPL*_*CT*_* in the modified PPL assay*

**Figure 7:**
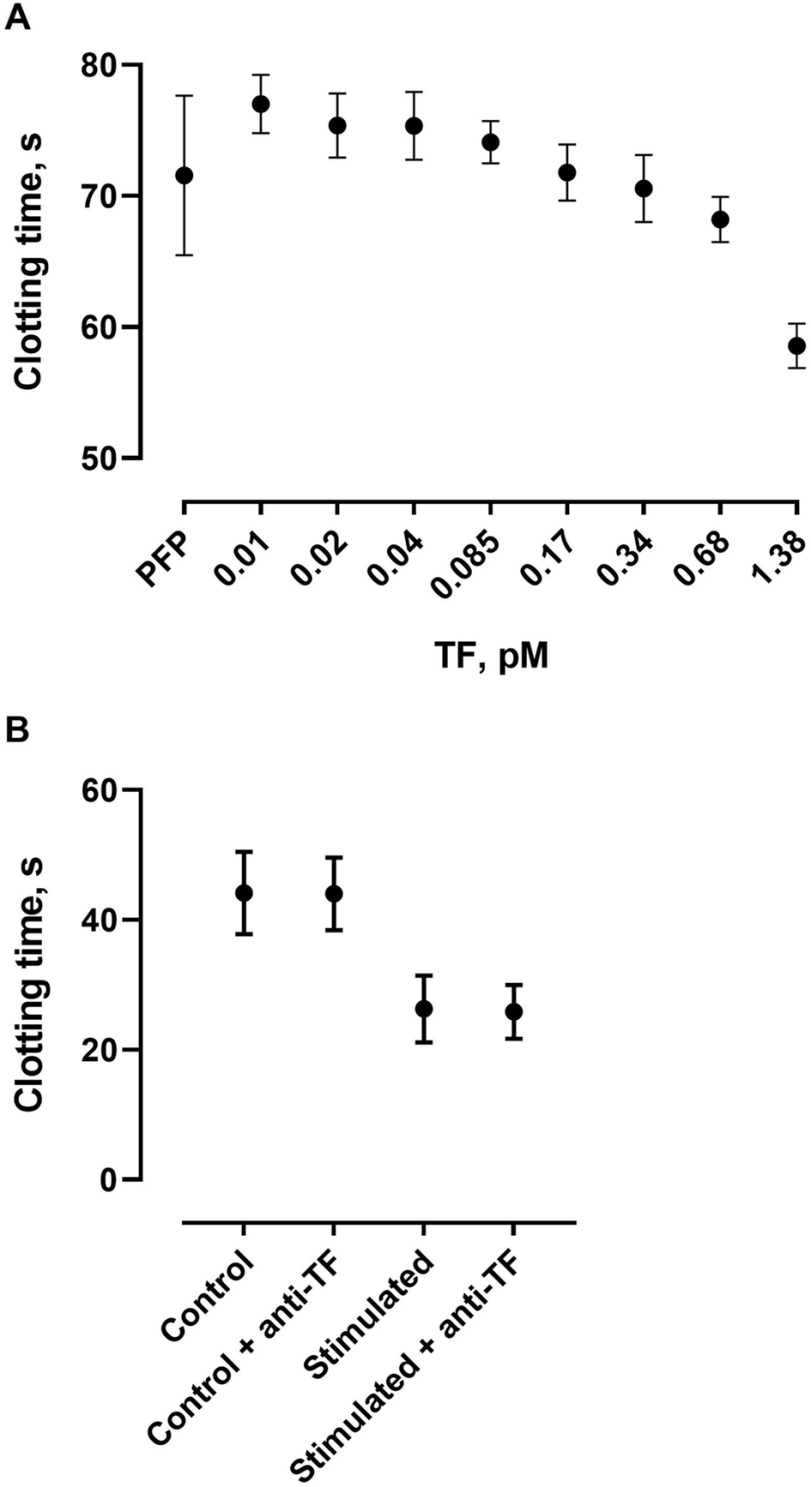
The effect of TF on the modified PPL assay. Exogenous relipidated TF was added to the modified PPL assay in concentrations from 0.01 to 5.5 pM (panel **A**). Panel (**B**) illustrates the effects of EVs from LPS-PMA stimulated blood and inhibitory TF antibody on clotting times in the PPL assay. Values are means ± 1 SD (panel **A**,**B**) from three experiments.

Addition of recombinant relipidated TF to PPL depleted plasma resulted in a dose-dependent shortening of clotting time (Fig. 7A). Since the artificial nature of TF and supra-pathological levels of TF may have caused the observed effect, we decided to test a more physiological source of TF. Whole blood was stimulated with a combination of LPS and PMA^12^ to induce the release of TF^+^ EVs from monocytes. Then, isolated EVs were run on the PPL assay with or without inhibitory TF antibody (clone HTF-1). As seen in Fig. 7B, stimulation of whole blood resulted in significant shortening of the clotting time. However, addition of anti-TF antibody (26 µg/ml final concentration) did not alter clotting times, suggesting that TF did not affect coagulation times in the modified PPL assay.

## Discussion

The procoagulant potential of plasma phospholipids is modified under pathological conditions. Currently, two commercial assays, namely XACT and STA-Procoag PPL are widely used to monitor PPL activity plasma. We thoroughly validated a modified and easy to use PPL assay for the measurement of procoagulant phospholipids in test specimens (plasma samples or isolated EVs) and compared its performance with the STA-Procoag-PPL assay. Our primary modification included preparation of PPL-depleted plasma by ultracentrifugation to remove EVs, assumed to be the main source of negatively charged phospholipids in plasma. This modification allowed for the establishment of an accessible and in-house assay with a comparable performance to that of the commercial assay. The addition of a standardized phospholipid reagent (UPTT) allows for clotting times to be converted into a standardized unit of phospholipids.

The commercially available PPL assays are well established in research and provide a reliable tool for assessing PPL activity in human plasma. Such assays are dependent on chemically phospholipid depleted plasma provided by the assay as a reagent. Their performance has been assessed under different pre-analytical and experimental settings such as quality control of cell storage^13^, plasma EVs^14,15^, and monitoring of plasma EV levels in various disease states^16–19^. Assuming that the vast majority of procoagulant phospholipids in plasma are EV-bound, we examined whether sequential centrifugation, including final ultracentrifugation, might substitute phospholipase treatment for production of a suitable assay plasma. Indeed, subjecting plasma to sequential centrifugation resulted in prolonged clotting times (i.e. reduced PPL activity), thus supporting that the major part of PPL activity in plasma is mediated by EVs, as originally suggested by Chargaff and West^3^.

Since ultracentrifugation may leave residual amounts of PPL in the assay plasma, it was crucial to compare the properties of our PPL depleted plasma with the phospholipase-depleted plasma. Therefore, the performance of the modified PPL assay and the STA-Procoag-PPL assay were compared using a standardized UPTT reagent. The results revealed a high degree of coherence between assays. Further, measurement of endogenous plasma PPL in PFP obtained from 10 volunteers demonstrated strong correlations between assays assessed by PPL_CT_. These findings support that the modified assay provide comparable performance to the STA-Procoag PPL assay with regard to sensitivity and measurement of levels of PPL in plasma.

It is important to note the effects of pre-analytical parameters on the performance of the modified PPL assay. Recently, Lacroix and colleagues used the STA-Procoag PPL assay to investigate the effect of different centrifugation protocols for PFP preparation on plasma PPL_CT_^14^. They found that PFP prepared according to the International Society of Thrombosis and Haemostasis (ISTH) recommended protocol (2500×*g* for 15 min twice) yielded similar PPL_CT_ to another common centrifugation protocol (1500×*g* for 15 min followed by 13,500×*g* for 2 min) in fresh PFP. However, PFP obtained by the second, less intensive centrifugation protocol, displayed a substantial shortening of the PPL_CT_ after a freeze–thaw cycle (− 154%). A minor effect on PPL activity (− 5%) was observed when analyzing frozen-thawed PFP using the ISTH recommended centrifugation protocol^14^. Accordingly, we found that a routine protocol for PFP preparation (3000×*g* for 10 min followed by 13,500×*g* for 2 min) yielded a 24% shorter PPL_CT_ than PFP prepared as recommended by ISTH. Hence, the plasma centrifugation protocol should be taken into consideration when comparing plasma PPL_CT_ between studies. As plasma depleted of PPL is an essential reagent in the modified assay, the importance of not introducing assay variations is critical. We demonstrated that three independent batches of PPL depleted plasma prepared from the same donors performed similarly, supporting the use of ultracentrifugation as a method for PPL depletion.

An additional pre-analytical challenge for population based studies is the availability of fasting blood samples as the PPL activity in plasma may be affected by plasma levels of triglyceride-rich lipoproteins. We therefore tested the PPL activity in plasma isolated before (0 h) and 4 h (4 h) after a high fat meal on the modified PPL assay. PPL_CT_ was unchanged in fasting and postprandial plasma, suggesting that PPL_CT_ is independent of postprandial lipemia and that there is no need to use fasting blood samples to obtain reliable PPL activity in plasma samples. Similar studies on the effect of lipemia should be repeated using more stringent pre-analytical conditions of plasma collection to see whether lipemia has a slight albeit detectable effect on the procoagulant properties of EVs. However, our findings are supported by Silveira et. al. who reported no effect of postprandial lipemia on the overall PPL measures, using the STA-Procoag PPL assay^20^. Using the same assay, Mørk and colleagues showed that even a non-standardized meal and a shorter time interval between the ingestion of the meal and blood draw (75 min) resulted in no change in PPL between fasting and postprandial samples^21^.

Procoagulant phospholipids, and PS in particular, affect the activity of both the intrinsic and extrinsic tenase and prothrombinase complexes, as well as the activation of FXI by thrombin^6^. The inter-individual variability may affect the degree of activation of several coagulation factors in the test samples. We therefore tested a wide range of FVIIa and FVa concentrations, where high concentrations of coagulation factors proved to shorten the clotting time in the modified PPL assay. However, these effects occurred only at supra-physiological concentrations significantly higher than those observed in vivo*.* Another aspect is the presence of TF. While only minute quantities are normally present in human plasma, TF is thought to be a major procoagulant factor found in EVs^22^. Previously, Connor and coworkers showed that increasing concentrations (0–0.1%) of TF added to whole blood were insensitive to the XACT assay^4^. Accordingly, we found that monocyte-derived EVs expressing TF after LPS stimulation (pathophysiological conditions) didn’t affect the clotting time in our assay. However, we observed a dose-dependent decrease in the clotting times with increasing supra-physiological concentrations of relipidated TF. Taken together, our findings suggest that the modified PPL assay is not influenced by physiological concentrations of the clotting factors in the test samples.

Over the last decade the interest in assays measuring the negatively charged phospholipid fraction of plasma has increased along with the growing interest and knowledge about EVs. Elevated levels of EVs, most frequently measured by flow cytometry, were found in venous thromboembolism^23–25^, arterial cardiovascular diseases^25,26^, cancer^27,28^, atherosclerosis^29,30^ and diabetes^31^. In vascular disorders, the procoagulant properties of EVs are of particular interest. It has been shown that PS is the main phospholipid contributing to the procoagulant function^32^.

The abundance of PS on the EV surface is often used to characterize EVs, by exploiting the ability of annexin A5 or lactadherin to bind PS on the outer leaflet of the membrane. Lactadherin is a small glycoprotein that binds PS in a calcium-independent manner and with higher affinity than annexin A5^33^. It has been demonstrated that lactadherin is an effective anticoagulant blocking the activity of PS, and inhibits the procoagulant activity of blood cells, endothelial cells and extracellular vesicles by 80%^34^. Here we showed that PPL_CT_ was prolonged with increasing concentrations of lactadherin to the extent that it was no longer measurable in the assay, implying that the measured PPL activity is largely dependent on PS in the test samples. Similarly, using the XACT assay Aung et al. showed that pre-treatment of packed red blood cell supernatants by lactadherin prolonged clotting times^13^.

Any assay designed for large-scale applications should be reproducible over time, and it should be possible to compare the results between different laboratories. To solve the latter challenge we propose the introduction of the UPTT reagent—an inexpensive standardized preparation of rabbit brain cephalin, which allows for clotting times to be converted into a standardized unit of phospholipids. The XACT assay solves this problem by inclusion of a synthetic PPL calibrator, while the Stago assay leaves it up to the users to create a reference range and standards for the clotting time^35^. Further, our modified PPL assay displayed minor variation in the assay performance. CVs obtained with either the standards from the Stago STA-Procoag-PPL or in-house pooled PFP range from 2.8 to 4.1%, well within recommended acceptable limits for within-day and between-day variability. Similar results were shown by van Dreden and colleagues in the XACT assay, with intra-assay CVs of 3.3% and 3.1% for normal pooled plasma and patient plasma, respectively, and inter-assay CVs of 3.9% and 4.2%^10^.

There are two main considerations with the modified PPL assay. First, the results will be influenced by the presence of lupus anticoagulants as well as high concentrations of coagulation factors which may lead to falsely prolonged or shortened clotting times. This is common for all plasma-based assays, and should be accounted for when interpreting the results. The creators of the XACT assay tried to overcome this issue by using plasma of porcine origin. However, while it significantly decreased the assay sensitivity to some of the lupus anticoagulants, it failed to completely eliminate the problem^35^. Second, pre-analytical conditions and inter-individual variations might impact plasma concentrations of coagulation factors in PPL depleted plasma. However, our modified assay seems to be unaffected by variations within the pathophysiological range.

In conclusion, the use of sequential centrifugation, including final ultracentrifugation, to deplete plasma of procoagulant phospholipids performed equal to enzymatic depletion of phospholipids from plasma in a FXa-based clotting assay to determine PPL clotting times. In addition, we introduced a standardized PPL reagent (UPTT) which allows for clotting times to be converted into a standardized unit of phospholipids. These modifications allowed us to establish an accessible and convenient in-house assay.

## Materials and methods

### Study subjects and sample preparations

For the calibration of the modified PPL assay, blood was drawn from healthy volunteers (n = 25) aged 25–79 years old, by venipuncture of an antecubital vein using a 21-gauge needle and minimal stasis. Blood was collected into 3 mL tubes containing 3.2% sodium citrate (0.109 M, 1:9 v/v) (Vacuette^®^, Greiner Bio-One, Kremsmünster, Austria), 3 mL K2EDTA Vacuette^®^ tubes (Greiner Bio-One, Kremsmünster, Austria) for cell count or Fragmin (Sigma-Aldrich, St. Louis, Missouri, USA) for the Ca-ionophore experiments (preparation of EVs from PRP). The first 3 mL of blood were discarded. Blood was mixed with anticoagulant by gentle inversions of the tube. The samples were kept at room temperature (20–24 °C) and processed within 15 min of collection. Blood was centrifuged twice at 2500×*g* for 15 min to obtain platelet free plasma (PFP). Both individual PFP samples and pooled PFP (n = 11) were prepared. PFP samples were aliquoted and stored at − 80 °C until use.

Procoagulant phospholipid depleted plasma (PPL depleted plasma) was prepared by sequentially centrifuging citrated blood (n = 18) using the following protocol: 2500×*g* for 15 min twice followed by 100,000×*g* for 60 min at 16 °C (Beckman Optima LE-80 K Ultracentrifuge, rotor SW40TI, Beckman Coulter, Indianapolis, Indiana, USA). Supernatants were pooled, aliquoted and stored at − 80 °C until further use.

Activated partial thromboplastin time (aPTT) and prothrombin time (PT-INR) were determined for the pooled PPL depleted plasma used in the modified assay. Both aPTT and PT-INR were measured on ACL TOP 750 CTS (Instrumentation Laboratory, Bedford, MA, USA), using the kits SynthASil (Instrumentation Laboratory, Bedford, MA, USA), and STA-SPA + (Diagnostica Stago, Asnières sur Seine Cedex, France), respectively.

The reproducibility of three independent preparations of PPL depleted plasma from the same six donors were tested. Citrated PFP from six volunteers were centrifuged at 100,000×*g* for 60 min at 16 °C, pooled, aliquoted and frozen at − 80 °C. PPL_CT_ was measured using the modified PPL assay. Clotting tests were perform in PPL depleted plasma alone, or with UPTT added to mimic normal CT (1:3200 U/ml UPTT) and short CT (1:100 U/ml UPTT).

In order to investigate the impact of postprandial lipemia, forty study participants donated blood for plasma and serum analysis in a previously described study^11^. Briefly, blood was drawn from an antecubital vein using a 19-gauge needle in a Vacutainer system with minimal stasis in the morning after 12 h fasting and then 2, 4, 6, and 8 h after a standardized high fat meal (1 g fat/kg body weight). Blood for plasma preparation was collected into 4.5-mL Vacutainers (Becton Dickinson, Meylan Cedex, France) containing 0.129 M sodium citrate (1:9 v/v). Serum was prepared by letting blood clot for 1 h in a glass tube at room temperature. Plasma and serum were centrifuged at 2000×*g* for 15 min at 22 °C, transferred into cryovials (Greiner Labortechnik, Nürtringen, Germany) and stored at − 80 °C until further analysis. Blood samples collected before (fasting) and 4 h after the meal were selected for the present study. For analysis, samples were thawed, centrifuged 13,500×*g* for 2 min and measured on the modified PPL assay. Informed written consent was obtained from all participants, and the regional committee for medical and health research ethics (REC North) approved the study. The study was conducted in accordance with relevant guidelines and regulations.

### Assay reagents

The UPTT reagent was purchased from BioData Corporation (Horsham, Pennsylvania, USA). Bovine FXa (bFXa), FVIIa, bovine FV/Va (bFV/Va) were purchased from Enzyme Research Laboratory (South Bend, Indiana, USA). Human recombinant tissue factor was purchased from Sekisui Diagnostics, LLC (Stamford, Connecticut, USA) and lactadherin from Haematologic Technologies Inc. (Essex Junction, Vermont, USA). The STA-Procoag-PPL assay was purchased from Diagnostica Stago Inc. (Asnières sur Seine Cedex, France). All other chemicals were from Sigma-Aldrich (St. Louis, Missouri, USA).

### Test procedure for the modified PPL assay

Clotting tests were carried out in duplicate by using a Start Max instrument from Diagnostica Stago (Asnières sur Seine Cedex, France). Twenty five µl of test plasma or EV suspension was mixed with 25 µl of PPL depleted plasma in a Start-cuvette containing a steel ball, and pre-warmed for 2 min to 37 °C. The reaction was initiated by the addition of 100 µl of pre-warmed assay buffer with a cabled pipette that automatically starts the timer upon pipetting, and clotting time was measured. The assay buffer contains bFXa (0.1 U/ml) in 15 mM calcium chloride, 100 mM sodium chloride and 20 mM HEPES buffer (pH 7.0). The STA-Procoag-PPL assay (Asnières sur Seine Cedex, France) was performed according to the manufacturer’s protocol.

### Assay calibration

Citrated blood samples from six individuals were subjected to sequential centrifugation in order to remove an increasing amount of EVs. The centrifugation protocol was as following; 2500×*g* for 15 min, 2500×*g* for 15 min twice, 20,000×*g* for 30 min and 100,000×*g* for 60 min. Clotting time was measured after each centrifugation step.

Concentration of bFXa for the modified assay was determined by serially diluting bFXa from 0.01 to 2 U/ml. Clotting time was measured using the modified PPL assay. Pooled PFP added to PPL depleted plasma or PPL depleted plasma alone were used as test plasma.

Standardized UPTT reagent containing 0.1% of rabbit brain cephalin in a buffered solution was used as a calibrator. The UPTT reagent was reconstituted in milliQ water according to the manufacturer’s protocol, and set as 1 U/ml. To obtain a standard curve, serial dilutions of the UPTT reagent (1/50 to 1/12,800) in the assay buffer were added to phospholipid-depleted plasma. UPTT dilution curves were measured for both the modified PPL assay and STA-Procoag-PPL assay.

The effect of PFP preparation was tested using two different centrifugation protocols. Citrated blood samples from ten volunteers were split in two, one fractions was centrifuged 2500×*g* for 15 min twice (75,000*g* minutes) and the second fraction 3000×*g* for 10 min, followed by 13,500×*g* for 2 min (57,000*g* minutes). Clotting time was measured using the modified PPL assay.

### The effect of coagulation factor V, VII, and blocking surface PS on PPL activity

The effect of varying levels of coagulation factors Va and VIIa were tested on the modified PPL assay. The coagulation factors were added to the reaction buffer in concentrations from 0.02 to 3.0 nM for FVa, and from 5 to 80 nM for FVIIa, and tested in pooled PFP.

The effect of lactadherin pretreatment of PRP-derived EVs on PPL activity was tested on the modified PPL assay. Plasma EVs were isolated from pooled citrated PFP, diluted with PBS (without Ca^2+^ and Mg^2+^) 1:3 v/v and centrifuged at 20,000×*g* for 30 min at 22 °C. The supernatant was discarded and the EV-pellet was resuspended in PPL depleted plasma. PRP-derived EVs were prepared by incubation of PRP (anticoagulated with Fragmin) with 10 µM of calcium ionophore A23187 for 15 min at 37 °C, followed by the isolation procedure described above. The concentrated EV suspension was serially diluted with PPL depleted plasma and assayed within 1 h.

### *The effect of TF and TF* + *EVs on PPL activity*

Recombinant relipidated TF was added in the reaction buffer to pooled PFP in concentrations from 0.01 to 1.38 pM and clotting time was measured on the modified PPL assay.

Whole blood was stimulated with a combination of LPS (5 ng/ml, Dako, strain 026:B6, Difco Lab., Detroit, MI, USA) and PMA (30 ng/ml, Sigma-Aldrich, Oslo, Norway) at 37 °C for 4 h with gentle agitation. EVs were isolated as described above. TF activity was blocked with an inhibitory antibody at a final concentration of 26 µg/ml (Purified Mouse Anti-Human CD142, Clone HTF-1, Catalog No.550252, BD Biosciences, Pharmingen, NJ). Clotting time was measured on the modified PPL assay.

### Serum lipid analysis

Serum triglycerides were measured by the use of an enzymatic photometric method on the ABX Pentra 400 instrument (Horiba ABX Diagnostics, Montpellier, France).

### Statistical analysis

Statistical analysis were performed in Graph Pad Prism 9.0.0 (GraphPad Software, Inc. La Jolla, CA, USA). ANOVA was used to test for differences in performance between the modified PPL assay and the STA-Procoag- PPL assay.

## Supplementary Information

Supplementary Information
