# Supplementary material for: A modified clot-based assay to measure negatively charged procoagulant phospholipids
Source: Sci Rep. 2021 Apr 29;11:9341. doi: 10.1038/s41598-021-88835-y (PMC8085201; doi:10.1038/s41598-021-88835-y)
Supplement: Supplementary file 1 — Supplementary Information [file 41598_2021_88835_MOESM1_ESM.pdf]

A modified clot-based assay to measure negatively charged procoagulant phospholipids

C. Ramberg[1,*], S. Jamaly[1], N. Latysheva[1], L. Wilsgård[1], T. Sovershaev[1], O. Snir[1], J.-B. Hansen[1,2]

[1] K.G. Jebsen Thrombosis Research and Expertise Centre (TREC), Department of Clinical Medicine, UiT – The Arctic University of Norway, Tromsø, Norway

[2] Division of Internal Medicine, University Hospital of North Norway, Tromsø, Norway

Supplementary Figures

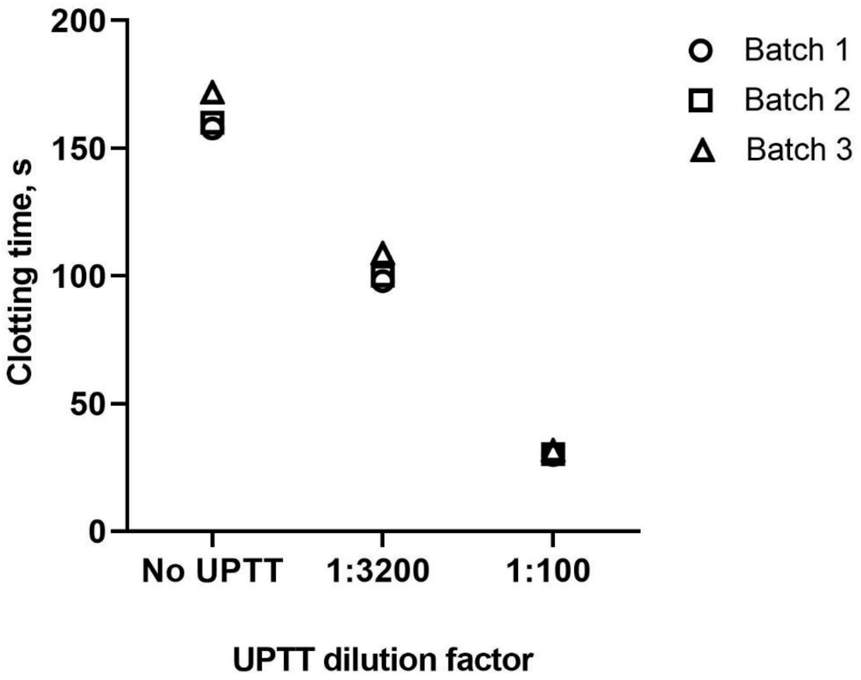

Supplementary Figure S1. The batch-to-batch reproducibility of EVDP. Citrated blood samples from six volunteers were centrifuged twice at 2500xg for 15 minutes, then 100,000xg for 1hour to prepare EVDP. EVDP samples were pooled and stored at -80°C until analysis. Three separate batches were prepared and the clotting times (CT) were measured using the modified PPL assay for EVDP alone, and with the addition of UPPT concentrations of 1:3200 (normal range CT) and 1:100 (short range CT). Values are mean of duplicate measurements ± 1 SD.

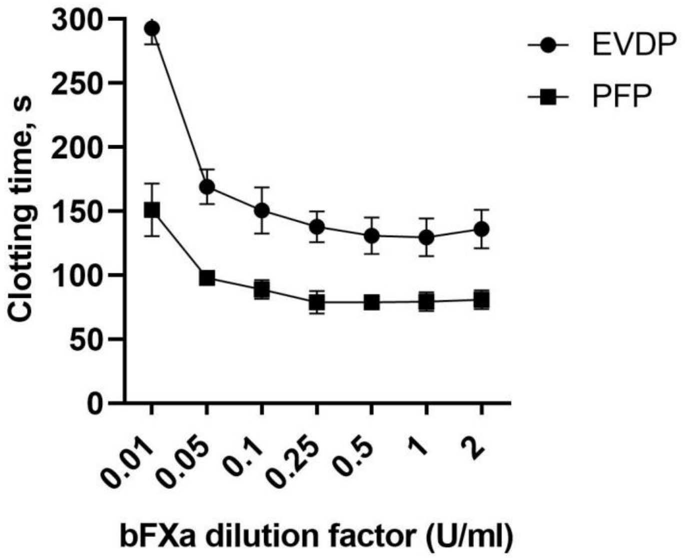

Supplementary Figure S2. Bovine FXa dilution curve. Clotting times were measured from serial dilutions of bFXa added to EVDP alone, or in combination with pooled platelet free plasma (PFP). Clotting times for EVDP alone using 0.01 U/ml bFXa exceeded the range of the instrument (300 s) and artificial values of 301 seconds were plotted. Values are mean of three experiments ± 1 SD.
